# Universal Health Checkups and Risk of Incident Diabetes and Hypertension

**DOI:** 10.1001/jamanetworkopen.2024.51813

**Published:** 2024-12-20

**Authors:** Masato Takeuchi, Tomohiro Shinozaki, Koji Kawakami

**Affiliations:** 1 Department of Public Health, Shizuoka Graduate University of Public Health, Shizuoka, Japan; 2 Department of Pharmacoepidemiology, Graduate School of Medicine and Public Health, Kyoto University, Kyoto, Japan; 3 Department of Information and Computer Technology, Faculty of Engineering, Tokyo University of Science, Katushika-ku, Tokyo, Japan

## Abstract

**Importance:**

Type 2 diabetes and hypertension are obesity-related, noncommunicable diseases becoming increasingly common worldwide. In 2008, Japan launched a nationwide universal health checkup program, Specific Health Checkup (SHC), for the primary prevention of obesity-related diseases, but its effectiveness has been understudied.

**Objective:**

To investigate the association of the SHC program with incident diabetes and hypertension, using a target trial emulation framework.

**Design, Setting, and Participants:**

This retrospective cohort study used data from a longitudinal health care database involving both checkup history and medical encounter records in Japan. Individuals aged 40 to 74 years, without diabetes or hypertension, and without a prior checkup history, were eligible. Individuals were repeatedly assessed for eligibility from April 1, 2008, to March 31, 2020, to assemble sequential cohorts of 78 620 SHC participants and 214 554 nonparticipants. Statistical analysis was conducted from June 8 to December 30, 2023.

**Main Outcomes and Measures:**

The composite risk of incident type 2 diabetes or hypertension over a period of up to 10 years, defined as the combination of a newly documented diagnosis and use of relevant medications. A propensity score–weighted survival analysis was conducted to adjust for baseline variables. A series of sensitivity analyses and a negative outcome control analysis were conducted using depression as a benchmark.

**Results:**

Sequential cohorts consisted of 78 620 SHC participants (median age, 46 years [IQR, 41-53 years]; 62.7% women) and 214 554 nonparticipants (median age, 49 years [IQR, 44-55 years]; 82.0% women) from 153 084 unique persons, each of whom entered the study cohort a mean (SD) of 1.9 (1.5) times. Within a median follow-up of 4.2 years (IQR, 2.7-6.3 years), the primary end point occurred among 11.2% of all individuals (10.6% of the SHC participants and 11.4% of the nonparticipants), with a lower hazard ratio (HR) among the SHC recipients (HR, 0.90; 95% CI, 0.89-0.92); the difference in cumulative incidence at 10 years was −1.6% (95% CI, −1.8% to −1.3%). The sensitivity analyses showed similar results. The negative control analysis suggested the potential for residual confounding (HR, 1.05; 95% CI, 1.02-1.07); the bias-calibrated HR was 0.86 (95% CI, 0.84-0.89) for the primary outcome.

**Conclusions and Relevance:**

In this cohort study, within a median of 4.2 years of follow-up, SHC recipients had a 9.8% lower risk of incident diabetes and hypertension (13.8% in the bias-calibrated analysis). The cost-effectiveness of the SHC and its transportability to other regions are unclear, requiring future investigations.

## Key Points

**Question:**

Is Japan’s universal health checkup system associated with primary prevention of obesity-related diseases, including diabetes and hypertension?

**Findings:**

In this cohort study using a target trial emulation framework to study 293 174 individuals, the risk of the composite end point of incident diabetes or hypertension was 9.8% lower among the recipients of the universal health checkup. A series of sensitivity analyses supported the robustness of the findings.

**Meaning:**

This study suggests that a universal health checkup system is associated with a lower risk of incident diabetes and hypertension, but the system’s cost-effectiveness and its transportability to settings outside Japan remain to be elucidated.

## Teaser

This cohort study uses an emulated target trial framework to investigate the association of a universal health checkup system with primary prevention of incident diabetes and hypertension.

## Introduction

Obesity is a key factor associated with the development of type 2 diabetes and hypertension,^1,2^ 2 diseases currently posing challenges to global public health. Evidence-based strategies for preventing obesity and related diseases are currently implemented as “action plans” in several countries,^3^ but few strategies are offered for the primary prevention of diabetes and hypertension on an individualized basis.^4^

In response to increasing health care costs for obesity-related noncommunicable diseases, Japan launched a universal health care program, the Specific Health Checkup (SHC), in 2008.^5,6^ Rather than secondary prevention or early detection of diabetes or hypertension among undiagnosed individuals, the primary purpose of the SHC is to identify individuals with high obesity-based risks of developing diabetes or hypertension (eTable 1 in Supplement 1), who are then invited to participate in lifestyle counseling if they are not currently undergoing pharmacologic treatment (eg, medications for diabetes and hypertension). Every citizen aged 40 to 74 years living in Japan is eligible for annual health checkups without incurring service charges. Participating in the SHC program is recommended by the government, but only approximately 50% of eligible individuals take advantage of this program.^6^ Although the SHC has been implemented as a public health policy for 15 years, its effectiveness as a primary prevention program has not been systematically evaluated.^7^ Randomized clinical trials (RCTs) are ideal solutions for addressing this open question, but such trials are not practical for ongoing nationwide health care programs.

Target trial emulation is a conceptual framework for nonrandomized studies to analyze observational data in a manner similar to RCTs.^8,9^ By leveraging the trial emulation framework, we aimed to evaluate the preventive association of the SHC program with incident type 2 diabetes and hypertension using a longitudinal health care database covering approximately 10% of the Japanese population.

## Methods

The original protocol was published elsewhere^10^; the changes from this original protocol implemented in the current study are documented in Table 1 and eAppendix 1 in Supplement 1. We followed the Strengthening the Reporting of Observational Studies in Epidemiology (STROBE) reporting guideline for cohort studies. Institutional review board approval was obtained from the Kyoto University Graduate School and Faculty of Medicine Ethics Committee. Given that we used anonymized data, consent from each individual was waived by the Kyoto University Graduate School and Faculty of Medicine Ethics Committee.

**Table 1.** Summary of the Target Trial Protocol and Emulated Protocol

Protocol component	Target trial (ideal RCT)	Emulation using observational data
Eligibility criteria	Persons aged 40-74 y^a^ No type 2 diabetes or hypertension No history of SHC participation or other types of health checkups^a^	Similar to the target trial Data lookback period ≥12 mo ≥1 Medical visit during the lookback period (within 365 d)
Treatment strategy	Receipt of SHC vs nonreceipt	Same as the target trial
Assignment procedure	Randomization	Randomization is emulated by nested trial design
Follow-up period	10 y Since randomization	Disenrollment from JMDC database (including death) Administrative end of the study 10 y After the enrollment in each emulated trial Outcome occurrence
Outcome	Composite of incident type 2 diabetes and hypertension^b^	Same as the target trial *ICD-10* codes and ATC codes were applied^c^
Causal contrast of interest	ITT principle Expected estimand: the association of being assigned to each intervention, irrespective of any postrandomization deviations (that can occur in both groups)	ITT principle^a^ Expected estimand: the association of being assigned to each intervention, irrespective of any postrandomization deviations (that can occur in the non-SHC group only)
Analysis	Cox proportional hazards regression model	Pooled logistic regression (adjusted with inverse probability weighting)

Abbreviations: ATC, Anatomical Therapeutic Chemical; *ICD-10*, *International Statistical Classification of Diseases and Related Health Problems, Tenth Revision*; ITT, intention-to-treat; RCT, randomized clinical trial; SHC, Specific Health Checkup.

^a^ Modified in the sensitivity analyses.

^b^ Changed from the original protocol.

^c^ For type 2 diabetes, *ICD-10* codes of E11 and E14 and ATC code of A10 were used. For hypertension, *ICD-10* codes of I10-I13 and E15 and ATC codes of C02, C03, and C07-C09 were used.

### Data Source

The data used in this study were extracted from the JMDC database, a longitudinal health care database provided by a commercial data vendor (JMDC Inc) that, since 2006, has collected data from more than 200 employee-based health insurance plans.^11,12^ The number of enrollees in the database has increased over time; as of 2019—the last year of the present study enrollment—the data from approximately 1.3 million individuals has been collected. In Japan, individuals aged 75 years or older are covered by a specialized insurance plan, and thus their data are not included in the JMDC database. Data regarding SHC records and medical encounters are stored in the JMDC database; both sets of information can be linked through a unique personal identifier. Compared with the general population in Japan, people in the JMDC database were primarily the employees of large companies and their family members. To minimize the computational burden, we randomly selected 20% of the individuals whose data were included in the JMDC database for this study.

### Target Trial and Emulation

Table 1 summarizes the core components of the ideal RCT (or target trial) and the emulating strategy. The included participants were sequentially screened to evaluate whether they met the eligibility criteria in each fiscal year (starting from April, in Japan), yielding a cohort comprising SHC participants and those who did not participate in the SHC at baseline^13^ (eFigure 1 in Supplement 1). The key inclusion criteria were as follows: aged 40 to 74 years; no diabetes or hypertension at enrollment; and no history of checkups, defined herein as individuals without a history of checkups, including by the SHC and other types of annual checkups offered as a voluntary service by insurance societies to those younger than 40 years. Other eligibility criteria included continuous enrollment in the JMDC database for at least 12 months and at least one instance of health care use in the preceding 12 months. The eligibility criteria were checked for all initially eligible participants yearly from April 1, 2008, to March 31, 2020, to form the nested cohort for each year; multiple cohort entries were allowed if the inclusion criteria were met in multiple fiscal years, as was the case in the non-SHC group.

### Sensitivity Analyses

We conducted 5 preplanned sensitivity analyses and 5 post hoc sensitivity analyses.

#### Preplanned Sensitivity Analyses

First, we conducted a per-protocol analysis to address nonadherence to the assigned intervention (ie, participating annual check-ups among the non-SHC group) using the inverse probability of censoring weight.^14^ Second, we included only persons aged 69 years or younger at study entry. Third, we also included persons who received checkups other than those from the SHC before cohort entry. Fourth, cohort entry was assessed monthly; due to the computational burden, 10% of the non-SHC samples were chosen randomly at each entry time point for this analysis. Fifth, the outcomes of diabetes and hypertension were evaluated separately; for this final sensitivity analysis, the inclusion criteria were modified to include persons without the index disease at baseline.

#### Post Hoc Sensitivity Analyses

The median follow-up period was approximately 4 years for the overall cohort (from 2008 to 2019), which was shorter than we expected. To ensure a 6- to 7-year follow-up period, cohort entry was limited from 2008 to 2016 in the first post hoc sensitivity analysis. Second, we entered the interaction term between SHC participant and time elapsed into the model; in this way, we could estimate hazard ratios (HRs) that may change over the course of follow-up. Third, we incorporated cumulative sessions of participating in the SHC since the time of cohort enrollment as a time-dependent covariate for the SHC group; for the non-SHC group, a constant value of zero was assigned for all time points.

Last, we performed negative control analysis,^15,16^ and explored selection bias and unmeasured confounding factors by using depression (*ICD-10* codes F32, F34, and F35) as a negative outcome.^17^ We also calculated the bias-calibrated HRs and E-values (eAppendix 2 in Supplement 1)^18,19^; these analyses were added to determine the potential residual bias suggested via negative control analysis.

### Statistical Analysis

Statistical analysis was conducted from June 8 to December 30, 2023. The primary outcome of interest was the composite of a diagnosis of incident type 2 diabetes or hypertension, as defined by the newly recorded diagnostic code and use of relevant medications. Participants were followed up until withdrawal from the insurance society (including death), occurrence of the outcome, or the administrative study end in fiscal year 2022, whichever occurred first.

The baseline characteristics are summarized with descriptive statistics. Between-group differences were assessed using the standardized mean difference which, unlike the *P* value, is insensitive to sample size^20^; a standardized mean difference less than 0.1 is generally considered as negligible imbalance. Summary statistics for SHC items such as body mass index (BMI; calculated as weight in kilograms divided by height in meters squared), blood pressure, glucose levels, and self-reported smoking status are described for the SHC participants in Table 2.

**Table 2.** Baseline Characteristics of Cohort

Variable	SHC group (n = 78 620)	Non-SHC group (n = 214 544)	SMD^a^
Age, median (IQR), y	46 (41-53)	49 (44-55)	0.33
Sex, %			
Female	62.7	82.0	0.44
Male	37.3	18.0	
Fiscal year of entry	2017 (2014-2018)	2016 (2014-2018)	0.02
Employed, %	58.0	26.2	0.68
Preexisting illness, %			
Allergic rhinitis	42.2	44.0	<0.1^b^
URI	41.3	43.5	
Vision problems	41.2^c^	40.9	
BMI, median (IQR)^d^	22.0 (19.9-24.4)	NA	NA
≥25, %	20.4	NA	
Waist circumstance, median (IQR), cm	79 (73-86)	NA	NA
Above threshold for obesity, %^e^	21.4	NA	NA
SBP, median (IQR), mm Hg^f^	116 (106-127)	NA	NA
Hemoglobin A_1c_, median (IQR), %^g^	5.4 (5.2-5.6)	NA	NA
Self-report smoking, %^h^	20.2	NA	NA

Abbreviations: BMI, body mass index (calculated as weight in kilograms divided by height in meters squared); NA, not available; SBP, systolic blood pressure; SHC, specific health checkup; SMD, standardized mean difference; URI, upper respiratory tract infection.

^a^ Absolute value presented. If <0.1, between-group difference is negligible.

^b^ For all pairwise comparison.

^c^ Include disorders of refraction and accommodation.

^d^ Missing for 0.04% of participants.

^e^ Threshold for obesity, 85 cm or more for males and 90 cm or more for females.

^f^ Missing for 0.2% of participants.

^g^ Missing for 12.7% of participants.

^h^ Missing for 1.9% of participants.

Individuals who did or did not participate in the SHC may have different characteristics; thus, we applied inverse probability weighting (IPW) to adjust for baseline differences^21^ including age at entry, sex, calendar year of enrollment, employment status,^22^ number of hospitalizations in preceding 12 months (categorized into 0, 1, and ≥2), number of health care visits in the preceding 12 months (categorized into 1-2, 3-5, 6-11, and ≥12 based on the rounded quartile range), and prior medical histories, represented by the corresponding 3-digit *International Statistical Classification of Diseases and Related Health Problems, Tenth Revision* (*ICD-10*) codes. Because the database lacked a specific label to indicate missingness for these variables, we assumed the absence of the condition if it was not recorded, except in the cases of age and sex, which were fully documented. According to the *ICD-10*–based comorbidities, histories of type 2 diabetes (*ICD-10* codes E10 and E14; prescription record of glucose-lowering medication also required), hypertension (*ICD-10* codes I10-I15; antihypertensive medication use also required), pregnancy-related conditions (*ICD-10* codes O.xx), and perinatal-specific diseases in children (*ICD-10* codes P.xx) were not used as covariates. Infrequent diagnoses (<0.01%) were also excluded because such codes were unlikely to contribute to the weight calculation and might introduce bias^23^ or induce computational inefficiency. The stabilized IPW was calculated and entered into the model to minimize the effects of extreme weights.^24^

The crude HR for the association between SHC participation and the composite outcome was determined with a univariable Cox proportional hazards regression model. Pooled logistic regression fitted with the stabilized IPW was applied to estimate the adjusted HR conditioned on baseline characteristics and the time since cohort entry (in years, converted to spline form).^25^ The primary analysis was performed on the basis of the intention-to-treat principle (ie, those who participated in the SHC later were not censored in the non-SHC group). The 95% CI was calculated by nonparametric bootstrapping with 1000 replicates; for some analyses with a high computational burden, the number of replicates was reduced to 200. We also estimated crude and adjusted differences in cumulative incidence between the SHC and non-SHC groups.^26^ All statistical analyses were done in R, version 4.3.2 (R Project for Statistical Computing).^27^

## Results

### Characteristics of the Study Cohort

**Figure 1.**
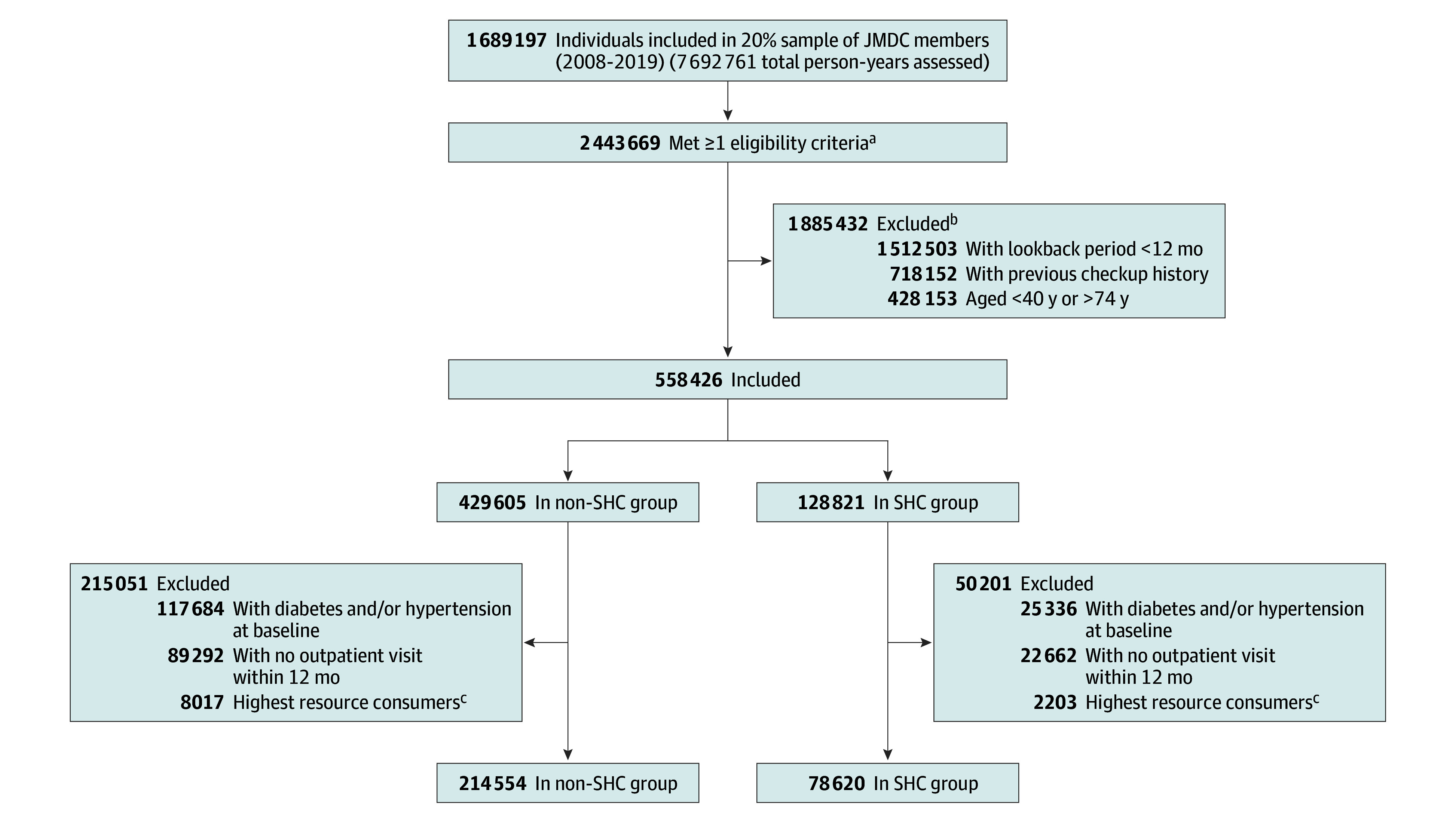
Flowchart of the Study Cohort ^a^SHC indicates Specific Health Checkup. ^a^Age between 40 and 74 years, no checkup history, and data lookback period of at least 12 months. ^b^One participant may fulfill several exclusion criteria. ^c^Proxies for physically handicapped individuals or nursing home residents, defined as those within the upper 99.99th percentile of resource use (outpatient visits and/or admission services).

From a 20% sample of all individuals in the JMDC database, we constructed sequential cohorts of 78 620 SHC participants (median age, 46 years [IQR, 41-53 years]; 62.7% women and 37.3% men) and 214 554 nonparticipants (median age, 49 years [IQR, 44-55 years]; 82.0% women and 18.0% men), for a total of 293 174 individuals (Figure 1 and Table 2); 153 084 unique persons were included in the cohort, each of whom entered the study cohort a mean (SD) of 1.9 (1.5) times. The most common preexisting illnesses in both the SHC and non-SHC groups were allergic rhinitis, acute upper respiratory tract infection, and disorders of refraction and accommodation. The median follow-up time up to 10 years was 4.2 years (IQR, 2.7-6.3 years); the most frequent reason for censoring (50.8%) was the study end in fiscal year 2022.

The characteristics of the study cohort are presented in Table 2. For the SHC group, the median BMI was 22.0 (IQR, 19.9-24.4) and the median waist circumference was 79 cm (IQR, 73-86 cm); such SHC-relevant data were not available for non-SHC group. Overall, 20.4% of SHC participants were classified as obese based on BMI and 21.4% were classified as obese based on waist circumference.

### SHC and Outcomes

**Figure 2.**
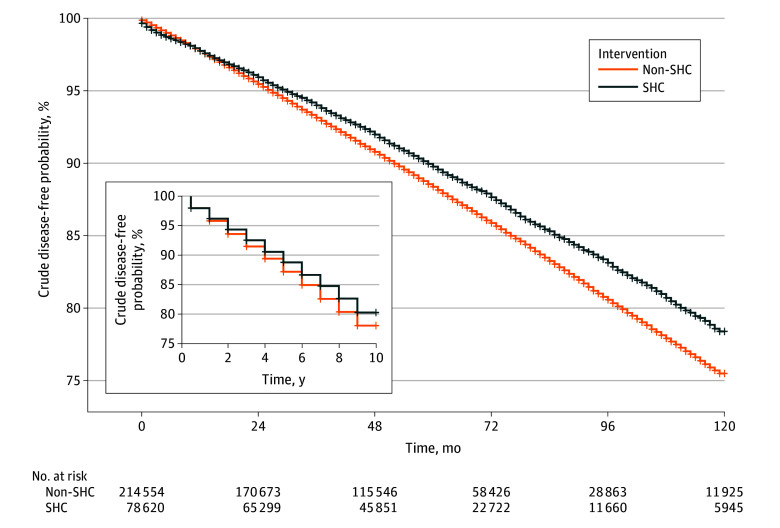
Crude Disease-Free Probability for Type 2 Diabetes and Hypertension Inset, survival curve in years. SHC indicates Specific Health Checkup.

The primary end point occurred in 11.2% of the enrollees during the follow-up period (10.6% of the SHC group and 11.4% of the non-SHC group). Figure 2 shows the unadjusted survival curves for the study population. The crude HR was 0.88 (95% CI, 0.86-0.91), and the difference in cumulative incidence at 10 years was −1.8% (95% CI, −2.0% to −1.6%).

**Figure 3.**
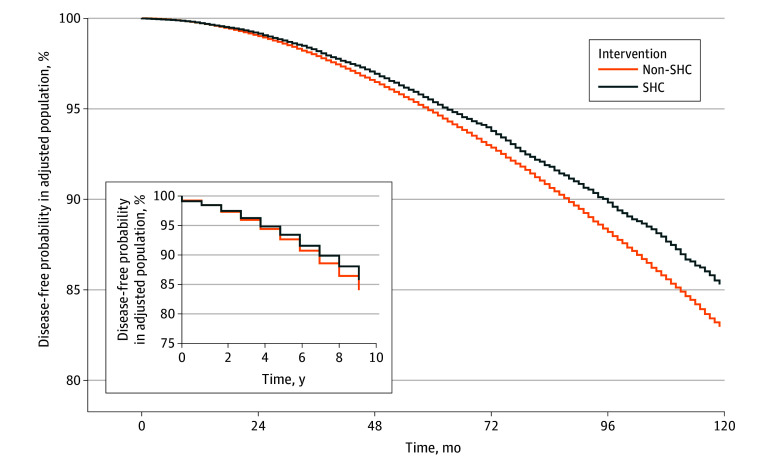
Disease-Free Survival Probability in the Population Adjusted by Inverse Probability Weighting Inset, survival curve in years. SHC, indicates Specific Health Check.

The IPW-adjusted survival curves are shown in Figure 3. Participating in the SHC program was associated with a lower risk of the composite outcome of type 2 diabetes or hypertension, with an adjusted HR of 0.90 (95% CI, 0.89-0.92), or alternatively, a difference in cumulative incidence at 10 years of −1.6% (95% CI, −1.8% to −1.3%). The distribution of propensity scores in each group is presented in eFigure 2 in Supplement 1.

### Sensitivity Analyses

The results of all sensitivity analyses favored the protective association of the SHC program (eTable 2 in Supplement 1). The adjusted HRs were 0.88 (95% CI, 0.87-0.90) in the per-protocol analysis in which, among 214 554 non-SHC participants, 67 811 eventually participated in the SHC, with a median follow-up time of 3 years (IQR, 2-4 years); 0.90 (95% CI, 0.89-0.92) in the analysis targeting persons aged 40 to 69 years; 0.90 (95% CI, 0.89-0.92) in the analysis that allowed cohort entry for persons who had already received other types of checkups; 0.96 (95% CI, 0.95-0.96) in the analysis that evaluated the eligibility criteria and outcome assessment on a monthly basis; 0.81 (95% CI, 0.78-0.84) in the analysis for incident type 2 diabetes; 0.92 (95% CI, 0.90-0.93) in the analysis for incident hypertension; 0.87 (95% CI, 0.86-0.88) in the analysis that limited the cohort entry until 2016, with a median observation period of 6.6 years (IQR, 3.7-8.7 years); 0.88 (95% CI, 0.87-0.90) in the analysis that encompassed nonproportional hazards; and 0.93 (95% CI, 0.91-0.95) in the analysis adding the covariate of the number of SHC sessions, with an HR of 0.94 (95% CI, 0.93-0.95) for each additional SHC participant.

As for the negative control analysis, the risk of developing depression was significantly greater among SHC participants (HR, 1.05; 95% CI, 1.02-1.07). The bias-calibrated HR was 0.86 (95% CI, 0.84-0.89) for the primary outcome, and the estimated E-value was 1.456 (95% CI, 1.048 to infinity).

## Discussion

We conducted a retrospective cohort study to investigate the preventive association of the SHC program in Japan using a large-scale longitudinal health care database. The study design and the analyses respected the principle of RCTs to the greatest extent possible, including the clear and ubiquitous definition of the “time-zero,” or the start of the follow-up point.^28,29^ This emulation study demonstrated that SHC participants had a 9.8% lower risk of incident diabetes and hypertension within a median of 4.2 years of follow-up. The negative control analysis suggested residual confounding, but this potential bias was unlikely to negate the beneficial association of SHC, as shown by the bias-calibrated HR and E-value.

Our findings appear somewhat contrary to results from previous RCTs and 1 meta-analysis of those RCTs concluding that general health checks are unlikely to be beneficial.^30,31^ One reason for this discrepancy may be due to differences in outcome definitions and study populations. For example, incident type 2 diabetes was an outcome of interest in our study, whereas it was regarded as a risk factor in a previous study.^31^ Overall, our study population was at low risk for future type 2 diabetes or hypertension because they were young and did not have diabetes or hypertension at baseline, in contrast to the findings of previous RCTs, in which 60% of participants were classified as high risk.^30^ Accordingly, our findings do not necessarily disagree with those of previous studies; rather, our study yielded novel insight into the effectiveness of a universal nationwide screening checkup program for the primary prevention of type 2 diabetes and hypertension.

The results of the present study could be promising for preventing a worldwide increase in the incidence of type 2 diabetes and hypertension. People with socioeconomic disparities, who are disproportionally at higher risk of obesity and related diseases,^32^ can also access this universal program. However, concerns regarding the cost may remain; the annual cost of the SHC program in Japan, which is funded by taxes and insurance fees, is estimated to be nearly $110 million US dollars.^33^ A cost-effectiveness analysis has yet to be formally conducted, partially due to the lack of objective data regarding the effectiveness of the program. Although cost-effectiveness was beyond the scope of our study, the present study provides quantitative data that can serve as the basis of a future cost-effectiveness analysis.

The transportability of our findings to settings outside Japan is also unclear. For example, the effectiveness of an obesity-centered checkup program may vary depending on the prevalence of obesity in each country, and the prevalence of overweight and obesity is relatively low in Japan.^34^ The direction of the association between the prevalence of obesity and the estimated effect size in this study is not predictable, unfortunately; thus, future studies are needed to examine the transportability of the program effect outside Japan.

### Limitations

This study has some limitations. First, it is susceptible to bias associated with residual confounding. Namely, preference for a healthy lifestyle, health care–seeking behaviors, or BMI can differ between participants and nonparticipants in the SHC, which in turn might confound our estimates of the association of the SHC program. To address this issue, we used hundreds of covariates to minimize baseline imbalances in the SHC and non-SHC groups. As a result, although a sign of early detection bias appeared in the unadjusted analysis (Figure 2), it was no longer observed after adjustment with IPW (Figure 3). We also conducted a negative control analysis and computed E-value, the results of which supported that bias alone cannot explain away a beneficial association of SHC program. Second, there is a potential for misclassification, as SHC participants could have been classified into the nonparticipant group because we were unable to access SHC records beyond the lookback period (a minimum of 365 days). Third, repeated sessions of the SHC may boost its protective association and prolong the duration of the preventive association, but such a dose–response association was not examined, as such an analysis has not been formulated in the trial emulation framework. One of the sensitivity analyses suggested a reduced risk of the composite outcome, with an HR of 0.94 for each additional SHC recipient, although these findings should be viewed as explanatory.^35^ Fourth, the mechanism behind the benefits of the SHC program was unclear partially due to the lack of data regarding who proceeded to the guidance program that was bundled with the checkup. One previous study reported that interventions via guidance programs were not efficient, and overall, attendance at guidance programs was low.^36^ As such, we assume that guidance programs alone were unlikely to be factors of the association of SHC in our case. Fifth, the results from target trial emulation did not necessarily agree with the findings from RCTs, even when carefully planned and conducted, attributable to such factors as residual bias or random error.^9^

## Conclusions

Our cohort study that emulated an RCT showed that within a median of 4.2 years of follow-up, SHC participants had a lower risk of incident diabetes and hypertension by 9.8%, or a lower cumulative incidence by 1.56% at 10 years. Future studies would be warranted as the cost-effectiveness and transportability to other settings outside Japan are uncertain.

## Notes

Supplement 1. **eTable 1.** Criteria for Educational Program (Specific Health Guidance) Invitation **eAppendix 1.** Changes From the Original Protocol **eAppendix 2.** Bias Analysis **eFigure 1.** The Illustrated Scheme of Sequential Cohort Entry **eFigure 2.** The Distribution of Propensity Score **eTable 2.** Details in the Sensitivity Analyses

Supplement 2.
Data Sharing Statement
