# Supplementary material for: Universal Health Checkups and Risk of Incident Diabetes and Hypertension
Source: JAMA Netw Open. 2024 Dec 20;7(12):e2451813. doi: 10.1001/jamanetworkopen.2024.51813 (PMC11662250; doi:10.1001/jamanetworkopen.2024.51813)
Supplement: Supplement 1. — eTable 1. Criteria for Educational Program (Specific Health Guidance) Invitation eAppendix 1. Changes From the Original Protocol eAppendix 2. Bias Analysis eFigure 1. The Illustrated Scheme of Sequential Cohort Entry eFigure 2. The Distribution of Propensity Score eTable 2. Details in the Sensitivity Analyses [file jamanetwopen-e2451813-s001.pdf]

Supplementary Online Content

Takeuchi M, Shinozaki T, Kawakami K. Universal health checkups and risk of incident diabetes and hypertension. *JAMA Netw Open.* 2024;7(12):e2451813.
doi:10.1001/jamanetworkopen.2024.51813

eTable 1. Criteria for Educational Program (Specific Health Guidance) Invitation
eAppendix 1. Changes From the Original Protocol
eAppendix 2. Bias Analysis
eFigure 1. The Illustrated Scheme of Sequential Cohort Entry
eFigure 2. The Distribution of Propensity Score
eTable 2. Details in the Sensitivity Analyses

This supplementary material has been provided by the authors to give readers additional information about their work.

© 2024 Takeuchi M et al. *JAMA Network Open.*

eTable 1. Criteria for Educational Program (Specific Health Guidance) Invitation

Obesity–related risk factor[1]	Checkup results	Program[2, 3]
**Waist circumstance**<br>≥ 85 cm (male)<br>≥90 cm (female)	• **BG**<br>fBG≥100 mg/dL *or* HbA_{1c} ≥5.6%<br>• **Lipids**<br>TG ≥150 mg/dL *or* HDL <40 mg/dL<br>• **BP**[4]<br>sBP ≥ 130 mmHg *or* dBP ≥85 mmHg<br>• **Self-reported smoking history**	• **Personal counseling and follow-up for 3 to 6 months**<br>If met ≥2 criteria<br>• **Personal or grouped counseling**<br>If met ≥1 criterion
**BMI**<br>≥ 25 kg/m^2	(same as above)	• **Personal counseling and follow-up for 3 to 6 months**<br>If met ≥3 criteria<br>• **Personal or grouped counseling**<br>If met 1 or 2 criteria

BMI: body mass index, BG: blood glucose, fBG: fasting blood glucose, HbA_{1c}: hemoglobin A_{1c}, TG: triglyceride, HDL: high density lipoprotein cholesterol, BP: blood pressure, sBP: systolic blood pressure, dBP: diastolic blood pressure

1: Essential requirement

2: Not offered to persons with current pharmacological treatment for hypertension, diabetes or dyslipidemia.

3: Invitation criteria for persons aged between 40 to 64 yrs; for those aged ≥65 yrs, counseling only.

4: In Japanese guideline, hypertension is defined as the threshold of 140/90 mmHg.

© 2024 Takeuchi M et al. *JAMA Network Open.*

eAppendix 1. Changes From the Original Protocol

- <u>Outcome definition</u>

We initially planned to write two separate research articles assessing diabetes and hypertension individually as primary outcomes because the SHC might affect each outcome differently. However, upon further consideration, we recognized that since the SHC was not specifically designed to prevent either condition exclusively, a composite of diabetes and hypertension would be the more appropriate outcome. Additionally, this approach would avoid the issue of 'salami slicing' in scientific reporting. Consequently, we opted to use a composite endpoint for diabetes and hypertension, with the individual outcome assessments for diabetes and hypertension being conducted as sensitivity analyses within this report.

This decision was finalized in August 2023, prior to conducting the formal outcome analysis.

- <u>Negative outcome control</u>

We formerly planned an analysis that included stomach cancer and brain tumors as negative control outcomes. However, during the data preparation, we noticed that these outcomes were infrequent, possibly because the study participants were young (median age at enrollment: 48 years). We consequently sought an alternative negative control outcome and selected depression. We assumed that the “U-comparability” assumption holds for depression based on our expertise knowledge and literature review. We verified that the number of new depression cases was comparable to the numbers of new diabetes and hypertension cases prior to proceeding with the formal analysis.

- <u>Sensitivity analysis incorporating the number of sessions the individuals participated in the SHC as an additional covariate</u>

We initially intended to use the cumulative number of SHC participations as a covariate in the main analysis. We observed that the HR was changed from 0.902 to 0.929 by adding this covariate. However, we also found that this covariate altered the HR for depression from 1.047 to 1.069, suggesting that this model could amplify the bias, as the SHC itself was unlikely to cause depression. Hence, we decided not to employ the cumulative number of participations in the SHC as a covariate in the primary analysis; instead, we reported the results of the corresponding sensitivity analysis.

© 2024 Takeuchi M et al. *JAMA Network Open.*

eAppendix 2. Bias Analysis

The concept of bias calibration was inspired by the article by Fu EL and colleagues (Eur Heart J. 2023 Jun 25;44(24):2216-2230); please refer to this article for further details.

We assumed that the strength of the relationship between the unmeasured confounders and the negative control outcome was equivalent to the strength of the relationship between the unmeasured confounders and the outcome of interest. Under this assumption, a point estimate of the bias-calibrated hazard ratio (HR) was calculated as follows:

$\exp(\log(0.902) - \log(1.047)) = 0.862$

The 95% confidence interval (CI) was calculated with 1000 bootstrapping samples, in which the HRs for the primary analysis and the negative control analysis were repeatedly calculated for each sample.

The E-value was computed via the evalues.HR function in the R::Evalue package (version 4.1.3).

In the above computation, we considered the direction of bias to be unidirectional, as the observed increase in the risk of depression among SHC recipients was unlikely to be causal. We additionally investigated an extreme case scenario in which the expected bias direction was completely opposite. In this case, the calibrated HR was 0.944 (95% CI: 0.918 to 0.972). As such, the protective effect of the SHC could not be explained away even in the extreme case (data not shown in the text body in the article).

© 2024 Takeuchi M et al. *JAMA Network Open.*

eFigure 1. The Illustrated Scheme of Sequential Cohort Entry

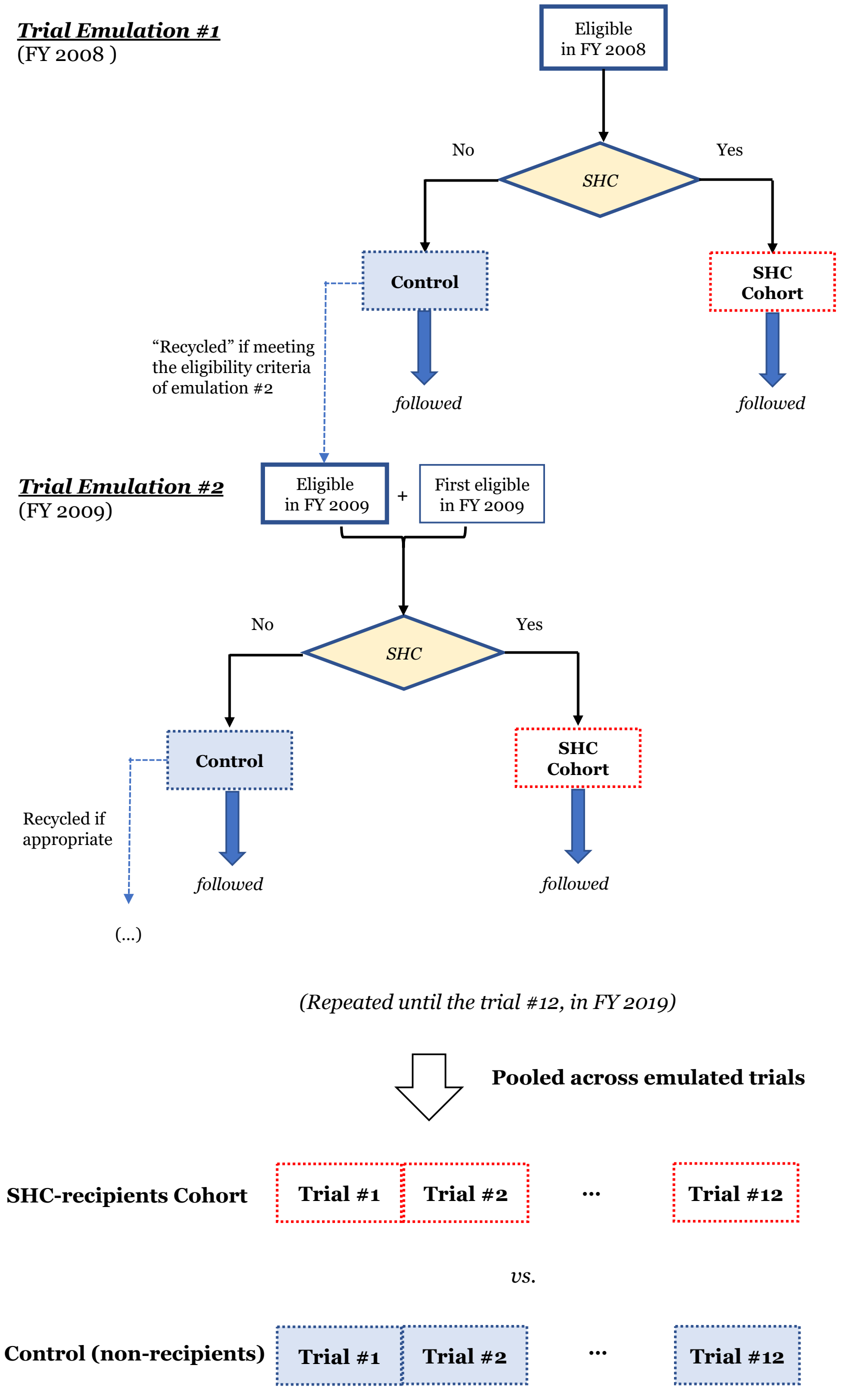

© 2024 Takeuchi M et al. *JAMA Network Open.*

eFigure 2. The Distribution of Propensity Score

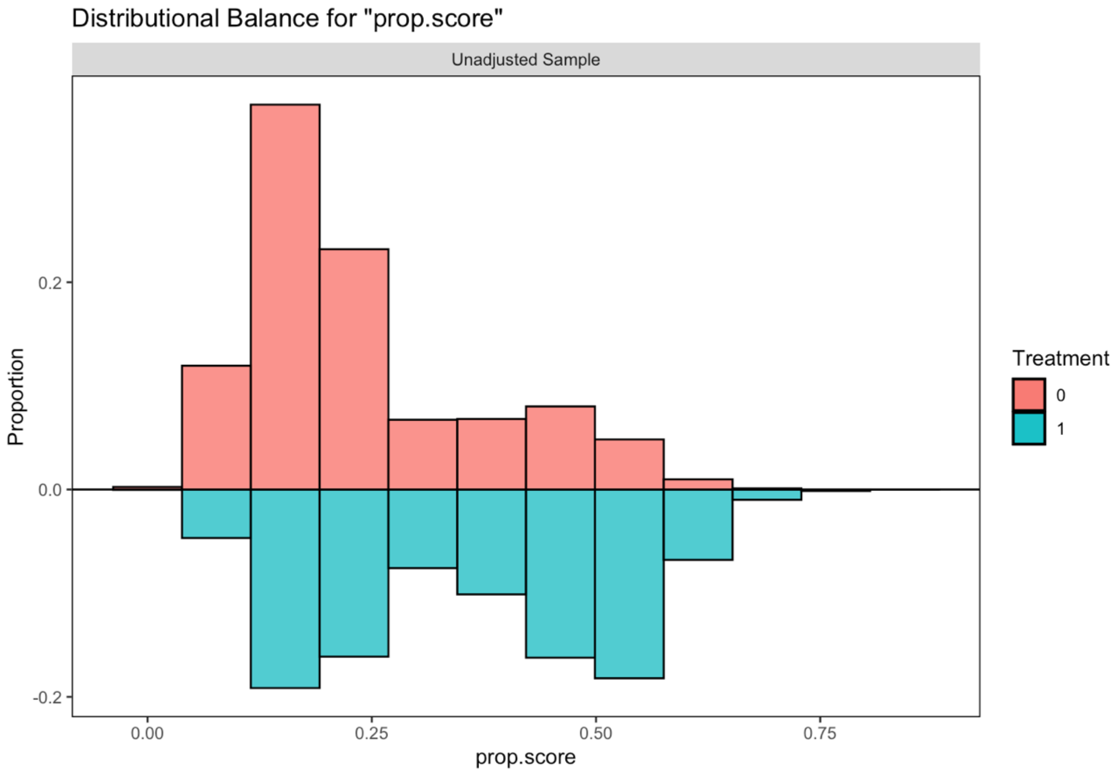

A propensity score represents the probability of receiving Specific Health Checkup (SHC). Treatment group one (teal blue) indicates the SHC group.

© 2024 Takeuchi M et al. *JAMA Network Open.*

eTable 2. Details in the Sensitivity Analyses

**Type of analysis**	**Cohort profile**	**aHR** (95% CI)
Per-protocol analys[1]	# of participant: 293 174<br>(26.8% in SHC group)<br># of outcome: 32 758	0.881 (0.866 to 0.895)
Persons aged 40 to 69 years	# of participant: 288 369<br>(27.0% in SHC group)<br># of outcome: 32 181	0.902 (0.888 to 0.916)
Included also persons with other type checkup history	# of participant: 293 026<br>(26.8% in SHC group)<br># of outcome: 32 739	0.902 (0.888 to 0.916)
Monthly-cohort entry[2]	# of participant: 395 436<br>(19.2% in SHC group)<br># of outcome: 43 059	0.958 (0.953 to 0.962)
Type 2 diabetes outcome[3]	# of participant: 293 026<br>(26.8% in SHC group)<br># of outcome: 5064	0.812 (0.784 to 0.840)
Hypertension outcome[3]	# of participant: 293 039<br>(26.8% in SHC group)<br># of outcome: 30 009	0.918 (0.904 to 0.933)
Entry period up to 2016	# of participant: 149 144<br>(26.1% in SHC group)<br># of outcome: 20 724	0.873 (0.857 to 0. 883)

© 2024 Takeuchi M et al. *JAMA Network Open.*

Non-proportional hazard case	# of participant: 293 174<br>(26.8% in SHC group)<br># of outcome: 33 758	0.881 (0.865 to 0.904)[4]
Additional covariate: cumulative number of receiving SHC[5]	# of participant: 293 174<br>(26.8% in SHC group)<br># of outcome: 33 758	0.929 (0.914 to 0.945)
Negative control analysis (depression)[6]	# of participant: 284 444<br>(26.8% in SHC group)<br># of outcome: 11 833	1.047 (1.021 to 1.073)
The primary analysis (reference)	# of participant: 293 174<br>(26.8% in SHC group)<br># of outcome: 33 758	0.902 (0.888 to 0.916)

SHC: Specific Health Checkup, aHR: adjusted hazard ratio (by inverse probability weighting), CI: confidence interval

1: Non-adherence was adjusted by inverse probability of censoring weight

2: For non-SHC group, 10% of random sampling was employed at each month (to prevent computational overflow)

3: Persons who had the index disease (diabetes or hypertension) at baseline were excluded.

4: Geometric mean up to 10 years

5: Added in SHC group only

6: Persons who did not have diabetes, hypertension and depression at baseline were enrolled.

© 2024 Takeuchi M et al. *JAMA Network Open.*
