# Supplementary material for: Universal Health Checkups and Risk of Incident Diabetes and Hypertension
Source: JAMA Netw Open. 2024 Dec 20;7(12):e2451813. doi: 10.1001/jamanetworkopen.2024.51813 (PMC11662250; doi:10.1001/jamanetworkopen.2024.51813)
Supplement: Supplement 2. — Data Sharing Statement [file jamanetwopen-e2451813-s002.pdf]

Data Sharing Statement

Takeuchi. Universal Health Checkups and Risk of Incident Diabetes and Hypertension. *JAMA Netw Open*. Published December 20, 2024. doi:10.1001/jamanetworkopen.2024.51813

Data
Data available: No

Additional Information
Explanation for why data not available: Data distribution is prohibited by data provider (JMDC Inc.)
